# Strategies for the Management of a Pulmonary Function Laboratory

**DOI:** 10.1016/j.chpulm.2024.100055

**Published:** 2024-04-06

**Authors:** Thomas W. DeCato, Matthew J. Hegewald

**Affiliations:** a Respiratory Medicine and Exercise Physiology, The Lundquist Institute, Harbor-UCLA Medical Center, Torrance, CA; b Division of Respiratory and Critical Care Physiology and Medicine, Harbor-UCLA Medical Center, Torrance, CA; c Department of Pulmonary and Critical Care Medicine, Intermountain Medical Center, Murray; d Division of Pulmonary and Critical Care Medicine, University of Utah, Salt Lake City, UT

**Keywords:** pulmonary function laboratory, pulmonary function testing, pulmonary lab

## Abstract

Pulmonary function tests (PFTs) are imperative to the diagnosis of people with respiratory symptoms and lung disease and to disease management. PFTs require expertise to obtain both high-quality tests and proficiency in test interpretation. In this review, we provide recommendations for obtaining high-quality test results in an efficient and cost-effective manner guided by available evidence and expert opinion. The medical director plays a critical role in pulmonary laboratory operations and ultimately is responsible for laboratory performance. Responsibilities of the medical director are reviewed and discussed. Quality control is an underappreciated part of the pulmonary laboratory that is that is necessary high-quality tests. What constitutes a complete PFT and the order that tests are performed may differ among laboratories. We suggest an approach to the performance of spirometry, bronchodilator-responsiveness testing, diffusing capacity, lung volumes, and tests of respiratory muscle strength that maximizes clinical usefulness and laboratory efficiency. Appropriate resources, time, and expertise are needed to run an efficient pulmonary function laboratory capable of performing high-quality testing.

## Introduction

Pulmonary function tests (PFTs) are a key component in the diagnosis of patients with respiratory symptoms and known or suspected lung disease and in disease management. The basic tests performed are: (1) spirometry (with or without bronchodilator administration), (2) lung volumes, (3) diffusing capacity, and (4) assessment of respiratory muscle strength. Additional useful laboratory tests include bronchoprovocation and exercise testing; these tests are not discussed in this review.

Optimal lung function testing requires two equally important components: technical expertise required to obtain high-quality results and expertise in the interpretation of the results. Our objective is to provide PFT laboratory medical directors and laboratory managers with strategies for obtaining the highest-quality results in an efficient and cost-effective manner. Informed by available evidence and expert opinion, we propose our approach for performing pulmonary function testing. The American Thoracic Society (ATS) Pulmonary Function Laboratory Management and Procedure Manual and the European Respiratory Society (ERS) and ATS technical standards for lung function testing are the primary resources aiding laboratory medical directors and managers in improving technical and procedural quality of pulmonary function testing.^1^, ^2^, ^3^, ^4^ Recommendations that diverge from the existing guidelines are presented and reasons for the differences are discussed. Interpretation of PFT results is discussed in detail by numerous other publications, most notably the recently published ERS and ATS technical standard on interpretive strategies for routine lung function tests.^5^

## Medical Director Responsibilities

Improperly performed testing and a lack of maintenance to instruments result in poor data that ultimately may contribute to incorrect diagnoses and therapy.^6^ The medical director should be a pulmonologist with expertise in PFT performance, laboratory management, understanding of the instruments used, and interpretation of results. Responsibilities of the medical director (Table 1) include ensuring the quality of the tests performed and that the interpreters are skilled and consistent in their readings.^1^^,^^7^ Specifically, interpretation should be consistent with updated guidelines. Variability in interpretation using % predicted, *z* scores, and fixed thresholds leads to confusion among referring providers. The medical director should review PFT interpretations periodically to confirm that interpretations are consistent with updated guidelines.^5^ Automated interpretation programs are available that may aid in accurate PFT intepretations.^7^^,^^8^ The laboratory medical director needs to develop a PFT report that is easy for referring providers to understand, with the results sent to the referring physician and entered into the medical record in a timely manner. Recommendations for the standardization of PFT reports are available.^9^

**Table 1:** Medical Director Roles and Responsibilities

• Creation of and updates to policies and procedures (ie, infection control; test performance; time allotted for testing; results into medical record, to referring provider, or both)
• Supervision and education of technical staff
• Availability to staff (ie, questions or concerns about testing)
• Oversight and analysis of quality control program
• Selection of reference equations
• Selection of tests performed and order of testing
• Recommendations and guidance on interpretive strategies to interpreting physicians

The laboratory medical director should provide continuous supervision and education of the technical staff. The director is responsible for the development of written policies and procedures that should be available for review in the laboratory.^1^^,^^6^ We recommend updating these in conjunction with ERS and ATS technical standards updates. Infection control policies and procedures are of particular importance, highlighted most recently by the COVID-19 pandemic forcing closure of laboratories. Time available to perform these tasks is necessary, although some tasks may need to be delegated to the laboratory supervisor/manager or to the chief technologist while providing oversight.

## Staff Training and Requirements

The ATS recommends that laboratory supervisors have a bachelor’s degree or higher in respiratory care or health care-related field, at least 4 years of experience in pulmonary function testing, and be a certified or registered pulmonary function technologist, with education or experience in business.^1^ Studies have shown that proper coaching by a skilled and knowledgeable technologist results in high-quality testing > 90% of the time.^6^ The ATS recommendation for a laboratory technologist is completion of secondary education and at least 2 years of college education.^1^ In North America, the National Board for Respiratory Care offers examinations to obtain additional credentialing (certified or registered pulmonary function technologist; www.NBRC.org/examinations/).

In general, standardized PFT laboratory technician training is lacking in the United States, with both variability in exposure among respiratory therapy students and on-the-job training. This again highlights why medical director oversight is imperative to ensure quality testing. The duration of training required to achieve competency is 6 to 12 months for common pulmonary function testing, whereas 1 to 2 years is recommended for troubleshooting instrumentation problems.^10^ Personnel performing testing as part of medical surveillance programs should attend further spirometry training approved by the National Institute for Occupational Safety and Health.^11^ Spirometry training does not guarantee high-quality testing and is particularly true in a primary care setting.^6^^,^^8^ Monitoring of technologist performance and technologist feedback improves the quality of testing, but is used by a minority of clinical laboratories.^12^ Currently no standards are available regarding staffing or production goals for laboratory technicians. An allotment of 60 min for so-called complete PFTs is reasonable. Also, no accreditation program is in place for pulmonary laboratories in the United States. Although the ATS previously worked on such a program, it is not currently active, although a laboratory can enroll in the ATS pulmonary laboratory registry (https://www.thoracic.org/professionals/pulmonary-function-testing/).^13^ The registry identifies pulmonary laboratories that are committed to following ATS and ERS standards for lung function testing.

## Quality Control

Quality control (QC) is the process of monitoring accuracy and precision of testing and instrumentation.^1^ Methodology includes using both biologic controls and mechanical models. QC includes 12 quality system essentials that give a framework for a well-managed laboratory and can be found in the ATS Pulmonary Function Laboratory Management and Procedure Manual.^1^ Activities such as training personnel, continued competency assessment, reporting results, record keeping, and maintaining and calibrating instruments are included in this system. The goal of QC activities is ensuring that measurements are within acceptable limits, reflecting the true or correct value and ensuring precision.

ATS and ERS guideline documents provide detailed discussion of calibration and QC requirements. Spirometry requires daily calibration verification at low, medium, and high flow with a 3-L syringe (some spirometer manufacturers state that their instruments do not require calibration, but all require verification). The 3-L syringe must undergo daily inspection, a monthly leak test, and volume verification as recommended by the manufacturer (typically yearly).^3^^,^^6^ At least monthly testing with a biologic control also is recommended. A biologic control ideally is a longstanding healthy nonsmoking member of the laboratory staff who can perform consistent lung function tests.

Single-breath diffusing capacity of the lung for carbon monoxide (Dlco) testing is a more complicated measurement requiring numerous specifications and performance standards for rapid gas analyzer systems used today. Gas analyzer calibration is performed before each test, along with daily volume calibration. Biologic control testing and a calibration syringe Dlco check are recommended weekly. A calibration syringe leak test and linearity check (calibration syringe or simulator) ideally are performed monthly. Further details can be found in the 2017 ERS and ATS standards for single-breath carbon monoxide uptake in the lung.^2^

QC for lung volume testing varies based on the method of testing. The recently published technical standard provides insight and detailed recommendations.^4^ For plethysmography, the flow-meter should be compliant with spirometry QC as noted. The mouth pressure transducer should be calibrated daily, although more frequent calibration may be needed if temperature or pressure are changing across the day.^4^ A flask with a thermal mass (eg, copper wool), referred to as an *isothermal bottle*, can be used for mechanical validation of accuracy. Both mechanical and biological QC should be performed monthly using two biologic control participants.^4^

**Figure 1:**
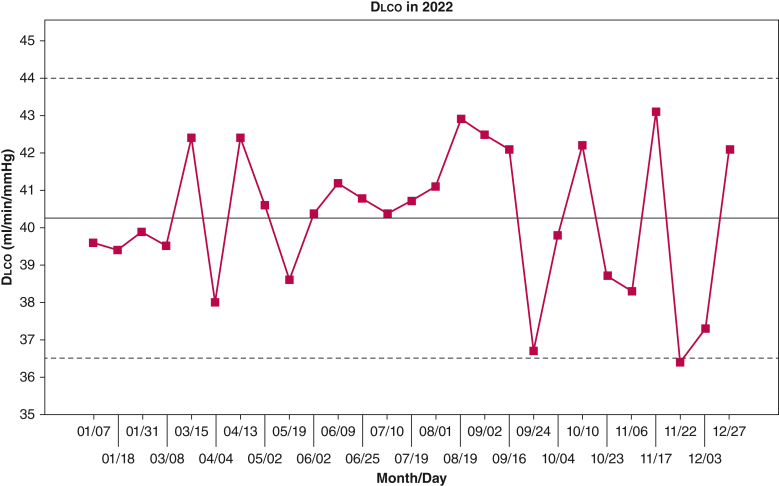
Levey-Jennings plot showing Dlco biologic control tests over 1 year. Hashed lines represent + and -1.96 z scores, whereas the solid line represents the mean. Dlco = diffusing capacity of the lungs for carbon monoxide.

After QC testing has been performed, analysis of the data must be completed. Because results follow a normal distribution, SD can be applied. With repeated testing, a database of mean values is established, followed by an SD. Westgard’s rules traditionally are applied to these data where both warning (± 2 SD) and out-of-control (± 3 SD) conditions can be identified using the SD from the mean of an individual value.^1^^,^^14^ Additional criteria exist for both warning and out-of-control conditions.^1^ Control charts or Levey-Jennings plots are a useful way to record and plot measurements. Figure 1 provides an example Levey-Jennings plot for Dlco tests performed using one of the author’s PFT instruments over 1 year. An Excel (Microsoft) program for recording spirometry, lung volumes, and Dlco results is included in the supplemental material. The medical director should be actively involved with the roles and responsibilities and ensure that QC testing is performed to current recommended standards. Our laboratories use *z* scores when evaluating QC data and consider results that deviate from a mean value by ± 1.96 *z* score to be out of range and prompt equipment inspection. This is a more stringent QC criterion than recommended by the ATS laboratory manual.^1^

A thorough QC program is a time-consuming process, but is essential for ensuring good-quality test results. Laboratory personnel must be allowed sufficient time for performing QC measures, and this will vary based on the size of a laboratory. Table 2^1^, ^2^, ^3^, ^4^ provides an overview of PFT laboratory QC procedures.

**Table 2:** Quality Control Procedures^1^, ^2^, ^3^, ^4^

Spirometer • Calibration according to manufacturers recommendation; some instruments do not require calibration. • If change in calibration factor is ≥ 6% or varies by more than ± 2 SD from the mean, inspect spirometer and calibration syringe. • All instruments require calibration verification; should be done daily and with change of flow sensor using a 3-L syringe at low, medium, and high flow with accuracy within ± 3.0%. • Recalibrate (if possible) and verify spirometer after failed calibration verification. • 3-L calibration syringe leak test monthly.
Dlco • Spirometer calibration and verification as stated. • Carbon monoxide and inert gas analyzer calibration per manufacturer recommendations; either daily or before each test. • Calibration syringe Dlco check weekly; 3 L test gas emptied into mouthpiece with measurement of VA (must be within 300 mL of 3 L times the standard temperature and pressure, dry (STPD) to body temperature and pressure, saturated (BTPS) correction factor) and Dlco (must be < 0.5 mL/min/mm Hg). • Gas analyzer linearity check with calibration syringe or Dlco simulator testing monthly.
Lung volumes (body plethysmography) • Mouth pressure transducer should be calibrated daily or before use. • Plethysmography signal should be calibrated daily using a volume signal of similar magnitude and frequency as the breathing maneuver during testing. • Accuracy validation using a known volume must be performed monthly, with new software, or if problem is suspected and undertaken using a model lung or container of known volume with accuracy within ± 50 mL or 3% (whichever is greater).
Biologic control testing • Performed weekly for Dlco and at least monthly for spirometry and lung volume measurements. • Record results in Excel data base (see e-Appendix 1 for example). • If *z* score is < –1.96 or > +1.96, inspect equipment, recalibrate spirometer or gas analyzer, compare results with other instruments (if available), consider changing flow sensor or gas analyzer.

Dlco = diffusing capacity of the lungs for carbon monoxide; VA = alveolar volume.

## Reference Value Selection

Ideally, the reference equations used would represent the population of the community served by the laboratory. Instrument software typically includes several equations, and studies have shown how the use of inappropriate reference equations can lead to both underdiagnosis and overdiagnosis, among other added financial and human costs.^6^ Use of race and ethnicity is such an example.^15^^,^^16^

Updated PFT interpretive strategies now recommend the use of Global Lung Initiative (GLI) reference equations for spirometry, Dlco, and lung volume testing (published in 2012, 2017, and 2022).^17^, ^18^, ^19^ These generally are believed to be the best equations published to date in part because of sample size, age range, and statistical method of derivation.^5^ Although now six GLI equations have been published for spirometry: four race-specific equations^17^ (White, Black, North East Asian, and South East Asian) and two race-neutral equations^17^^,^^20^ (Other and Global), it is now recommended to use a race-neutral equation. A joint ATS and ERS statement on the use of race and ethnicity in pulmonary function testing comprehensively reviewed the literature to provide current recommendations and future directions of study to better understand the role of socioenvironmental and genetic determinants of lung function.^21^ In brief, race is a social and not a biological construct, and using race-specific equations may perpetuate long-standing racial and health disparities.

The term *race-neutral* refers to an equation not requiring the selection of race for application. GLI Global equations are the most recent race-neutral equations published with the goal of reducing the bias associated with oversampling of White people, as done for GLI Other equations, in which 77% of the data came from this population.^17^^,^^20^ The overall difference between GLI Global and GLI Other equations is small (< 1% on average), although the SD is larger, with GLI Global resulting in the limits of normal being up to 9% wider for FEV_1_ and FVC.^20^ It should be noted that equations for both GLI Dlco and lung volumes were derived in White populations only.^18^^,^^19^ In-depth understanding of reference equations is required for application in the pulmonary laboratory, and our understanding of the differences in lung function between people continues to evolve. A laboratory should state the reference values used on the report. This is important when comparing results between laboratories or within a laboratory if the reference values have been changed (eg, National Health and Nutrition Examination Survey III to GLI). This may be particularly relevant for extremes of height and age, where differences between predicted spirometry values are larger.^22^

## What Is Complete Pulmonary Function Testing?

What constitutes complete pulmonary function testing has not been defined clearly and differs among laboratories. The ERS and ATS Technical Standard on Interpretive Strategies states that routine PFTs address three functional properties of the lung: airflow, lung volumes, and gas transfer.^5^ Complete PFTs for many laboratories include spirometry (before and after bronchodilator administration), lung volumes, and Dlco. Pulse oximetry often is included. Some PFT laboratories routinely measure maximum voluntary ventilation (MVV), maximal inspiratory pressure (MIP), and maximal expiratory pressure (MEP), measures of respiratory muscle strength. Complete PFTs including the above tests take approximately 60 min. It is possible to exclude some components of complete PFTs without affecting clinical usefulness, improving laboratory efficiency, decreasing costs, and improving the patient experience. However, we acknowledge that complete PFTs may differ between laboratories, depending on the clinical indication.

## Is Bronchodilator Responsiveness Testing Required for All?

Bronchodilator responsiveness (BDR) testing quantifies the improvement of airflow after the administration of a bronchodilator. The most common BDR protocol (2005 ATS and ERS spirometry standards) includes albuterol metered dose inhaler (100 μg per actuation) four puffs with a 15-min waiting time before postbronchodilator maneuvers.^23^ Administration of albuterol and ipratropium bromide will identify more patients with positive BDR, but the delayed onset of action of ipratropium bromide compared with albuterol requires a 30-min wait, decreasing laboratory efficiency.^23^^,^^24^

The 2019 ATS and ERS standards recommend that all initial spirometry performed for diagnostic reasons include bronchodilator administration. Subsequent testing may be performed without bronchodilator administration, although serial spirometry in obstructive lung disease may be “more useful following the post-bronchodilator values.”^3^ The 2023 Global Initiative for Chronic Obstructive Lung Disease report states that spirometry after bronchodilator administration is required for the diagnosis and assessment of COPD.^25^ The Global Initiative for Asthma 2023 update also recommends BDR testing to document variable airflow limitation.^26^ Despite these recommendations, the clinical usefulness of BDR testing has been questioned.^27^^,^^28^ The routine use of BDR testing should be scrutinized, because it adds a minimum of 20 min to spirometry and increases costs.

In patients with risk factors for COPD, from 7% to 27% will change from obstructed to normal spirometry findings after BDR testing, theoretically excluding a diagnosis of COPD.^29^^,^^30^ Although variable airflow limitation in asthma is diagnosed definitively with bronchoprovocation testing, positive BDR findings are useful for diagnosing asthma and are simpler and less costly than bronchoprovocation testing. BDR testing is of limited value in distinguishing asthma from COPD.^31^^,^^32^ BDR results are highly variable over time and are unreliable for phenotyping.^33^^,^^34^ BDR testing does not predict treatment response of long-term use of bronchodilators or inhaled corticosteroids in asthma or COPD.^35^^,^^36^ Although the updated ERS and ATS technical standards define positive BDR results as a > 10% increase in FEV_1_ or FVC relative to predicted value, a debate remains regarding what defines positive BDR results.^37^

Evidence shows that BDR testing can be excluded in some patients with a FEV_1_ to FVC ratio before bronchodilator administration within predicted limits and an FEV_1_ of > 100% predicted. Such patients are highly unlikely to exhibit positive BDR results and can forego BDR testing.^38^ Acknowledging that BDR testing may not affect clinical care in many patients, BDR testing may be most useful when performed as a component of a complete PFT protocol if any of the following criteria are present: spirometry findings before bronchodilator administration are abnormal; the patient is suspected of having asthma or COPD; or the patient has symptoms of dyspnea, cough, wheeze, or chest tightness (queried by PFT technicians). BDR testing is excluded if the FEV_1_ to FVC ratio is within predicted limits and the FEV_1_ is > 100% predicted.

## When Is Lung Volume Testing Necessary?

Methods for total lung capacity (TLC), functional residual capacity, and residual volume measurements include body plethysmography, nitrogen washout, and multibreath inert gas techniques.^39^^,^^40^ Lung volume testing is technically more challenging than spirometry, adding significant time to testing. Body plethysmography is considered the gold standard for assessing lung volumes because it includes poorly ventilated lung units.^4^ Nitrogen washout and inert gas techniques are acceptable alternatives when laboratory space is limited or patients have contraindications to plethysmography (eg, claustrophobia, BMI preclusions). Alveolar volume (VA) measured using a single-breath gas dilution technique in conjunction with Dlco testing, when combined with a measure of dead space, provides an approximation of TLC. However, VA systematically underestimates TLC with greater differences between VA and TLC in patients with inhomogeneous ventilation.^41^^,^^42^

Lung volume testing is required for the diagnosis of restrictive ventilatory impairment and to identify hyperinflation and air trapping.^5^ The main indication for performing lung volume testing is to confirm restriction in a patient with a low FVC. A low FVC and a normal FEV_1_ to FVC ratio may be the result of simple restriction, complex restriction, or the nonspecific pattern; each of these patterns then is determined by the TLC measurement.^5^ Similarly, in a patient with a low FVC and obstruction, lung volume measurement will help to determine if a concomitant restrictive process is present or if the low FVC is the result of air trapping. It must be stressed that a low FVC cannot diagnose a restrictive ventilatory impairment because only approximately 50% of patients with a reduced FVC will have a low TLC.^43^^,^^44^ Conversely, a FVC within normal limits can exclude a low TLC with high reliability because a normal FVC is associated with a low TLC in approximately 3% of patients.^43^^,^^45^ Because a normal FVC generally can exclude a restrictive ventilatory impairment, a number of studies have proposed forgoing lung volume testing to improve laboratory efficiency and to decrease costs.^46^^,^^47^ These studies have proposed various values for FVC % predicted combined with FEV_1_ to FVC ratio thresholds that predict a normal TLC. However, it may be feasible to use a simpler approach. If any measurement of vital capacity exceeds the FVC lower limit of normal, specifically a slow vital capacity, an inspired vital capacity, or an FVC (before or after bronchodilator administration), routinely performing lung volume testing may not add value. An inspiratory vital capacity should be performed routinely after a forced expiration as part of an FVC maneuver^3^ and also is measured during a Dlco test.^2^ Although slow vital capacity is not performed routinely except as part of lung volume testing, it should be considered in patients who do not meet end of forced expiration criteria^3^ and may be useful in older adult patients and those with obesity patients because slow vital capacity commonly is significantly larger than FVC in these patients.^48^ A protocol that included the largest vital capacity measured resulted in 15% of patients changing from a restrictive spirometry pattern (low maximal vital capacity) to normal spirometry findings, suggesting that restriction is unlikely, obviating the need for lung volume measurement.^49^

VA plus dead space approximates TLC in patients with homogeneous ventilation, but underestimates TLC in patients with parenchymal lung disease and airflow obstruction.^50^, ^51^, ^52^ Because a normal VA accurately predicts a normal TLC in the absence of obstruction,^51^ a protocol that omits lung volume testing if VA is within predicted limits, regardless of vital capacity result, may preclude the measurement of TLC because a restrictive ventilatory impairment is unlikely.

Although lung volume testing is required to diagnose a restrictive ventilatory impairment in a patient with a low vital capacity or low VA, body plethysmography is required to diagnose hyperinflation and air trapping. However, in patients with airflow obstruction, lung volume measurement adds little to clinical interpretation or management decisions.^53^ One notable exception is in patients evaluated for lung volume reduction procedures.^54^

We acknowledge that excluding lung volume testing, specifically with body plethysmography, a technique that provides a measure of airways resistance, in patients with normal vital capacity or VA may limit physiologic assessment. Performing lung volume testing may provide clinically important information when spirometry and Dlco results are near the lower limit of normal. Abnormal lung volume measurement in individuals who smoke with normal spirometry findings may predict morbidity and may identify patients at higher risk of COPD developing.^55^ Also, increased airway resistance or increased residual volume to TLC ratio in patients with normal FEV_1_ to FVC ratio may suggest a diagnosis of asthma or disease processes affecting small airways.^56^ However, we are not aware of studies showing that this information impacts management.^47^^,^^57^

## Should MVV Be Measured Routinely?

Assessment of respiratory muscle strength is important in the evaluation of neuromuscular disease, unexplained dyspnea, and unexplained decrease in FVC. Identifying respiratory muscle weakness predicts adverse clinical events and may lead to interventions improving outcomes. Serial testing is recommended to make a confident diagnosis because a single test may misidentify respiratory muscle weakness.^58^^,^^59^ Although no single lung function test will identify patients with respiratory muscle weakness adequately, so-called complete PFTs often include MVV as a screening test. The patient is instructed to breathe as deeply and quickly as they can for 12 to 15 s. The maximal ventilation in 12 s is calculated and multiplied by five. If MVV divided by FEV_1_ × 40 is < 0.80, the test results are abnormal.^23^ MVV, once touted as specific for respiratory muscle weakness, is highly effort dependent and is influenced by factors other than respiratory muscle function (airway resistance, respiratory system compliance).^23^^,^^58^ In patients without respiratory muscle weakness, MVV is reduced in proportion to vital capacity. Because it is a difficult and strenuous test to perform and adds little to more simple tests such as vital capacity, MVV should not be measured routinely as a component of complete PFTs.

## MIP and MEP

MIP and MEP are static pressures commonly used to measure maximal respiratory muscle strength. Although MIP and MEP are considered easy to perform and measure, it is important to consider that they are also volitional tests. Reference values are limited, and MIP and MEP should not be interpreted in isolation.^60^^,^^61^ Supine spirometry is another easily obtained measurement that should be considered when performing tests of respiratory muscle strength. Normally, the supine vital capacity decreases by 5% to 10% from the upright position. A decrease of 25% to 30% is associated strongly with diaphragmatic weakness.^58^^,^^62^^,^^63^ Performance of MVV, MIP, MEP, and supine spirometry all should be carried out in the assessment of respiratory muscle strength when respiratory muscle weakness is suspected.

## What Is the Preferred Sequence of Pulmonary Function Testing?

**Figure 2:**
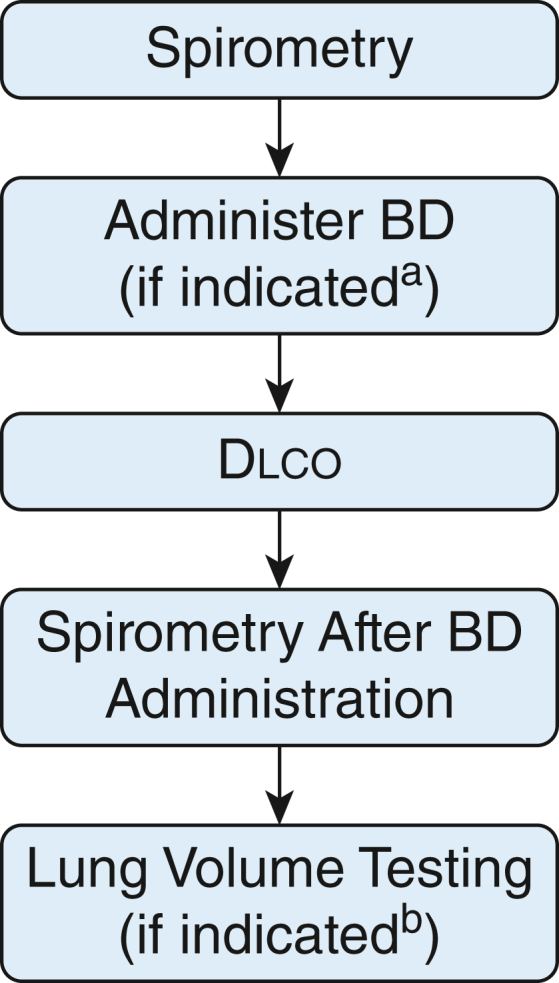
Flow diagram showing complete pulmonary function test protocol. ^a^Bronchodilator (BD) is administered if spirometry findings before BD administration are abnormal, patient has a diagnosis of asthma or COPD, or symptoms suggestive of asthma or COPD are present (dyspnea, cough, wheeze, chest tightness); BD is not administered if FEV_1_ to FVC ratio before BD administration is normal and FEV_1_ is > 100% predicted. ^b^Lung volume testing is not performed if any measure of vital capacity (maximum vital capacity) is normal or alveolar volume (obtained during diffusing capacity of the lungs for carbon monoxide testing) is more than the lower limit of normal. Dlco, diffusing capacity of the lungs for carbon monoxide.

A reasonable and cost-efficient complete PFT protocol may consist of: (1) spirometry with BDR testing (in selected patients), (2) single-breath Dlco, (3) lung volumes if vital capacity and VA are less than the lower limit of normal, and (4) pulse oximetry measurement. The order of test performance listed in Figure 2 maximizes laboratory and technician efficiency. If BDR testing is indicated, a bronchodilator is administered before the single-breath Dlco test. Single-breath Dlco measurement is not affected significantly by albuterol administration in healthy patients or in those with obstructive lung disease.^64^^,^^65^ Spirometry after bronchodilator administration is performed after single-breath Dlco and 15 min after bronchodilator administration and before lung volume measurements. The ERS and ATS technical standards on measurement of lung volumes recommends lung volume measurement before BDR testing.^4^ Deviating from the ERS and ATS recommendation may be considered, because a vital capacity measurement after bronchodilator administration in the normal range predicts a normal TLC with high likelihood, and therefore precludes the measurement of lung volumes in most situations.^39^^,^^41^ Bronchodilator administration may affect lung volume measurements, altering the severity of the assessment of hyperinflation and air trapping in patients with obstructive lung disease, but the clinical usefulness of static lung volume testing in these patients is questionable.^49^^,^^53^

## Conclusions

Running an efficient and effective pulmonary laboratory requires time and expertise. Test and reference equation selection, supervision and education of technical staff and colleagues, creation of policies and procedures, and a QC program are a few of the many important responsibilities under the purview of the medical director and laboratory staff. Assessing for bronchodilator responsiveness and lung volume testing may not be necessary for all patients and complete PFTs may vary depending on the patient. Details unseen by a casual look at the pulmonary laboratory are required to obtain high-quality testing and to result in clinically useful interpretation. This in turn requires appropriate time available and expertise of the medical director and technical staff.

## Funding/Support

T. W. D. is supported by the 10.13039/100000002National Institutes of Health [Grant R01HL166850].

## Financial/Nonfinancial Disclosures

The authors have reported to *CHEST Pulmonary* the following: T. W. D. has received consulting fees from MannKind Corporation and is an editorial board member of *CHEST* and *CHEST Pulmonary*.

## Supplementary Data

e-Online Data

e-Online Data
