# Supplementary material for: Strategies for the Management of a Pulmonary Function Laboratory
Source: CHEST Pulm. 2024 Apr 6;2(3):100055. doi: 10.1016/j.chpulm.2024.100055 (PMC13417573; doi:10.1016/j.chpulm.2024.100055)
Supplement: e-Online Data [file mmc1.docx]

**Excel spreadsheet instructions:**

Enter biocontrol results for the tests listed. A running mean value and z-score will be calculated. After 8 tests have been entered, a z-score exceeding +/- 1.96 will be displayed with a pink background indicating the result is “out of range.” This should prompt inspection of the instrument.
